# Nucleolus-like body of mouse oocytes contains lamin A and B and TRF2 but not actin and topo II

**DOI:** 10.1186/s13039-016-0259-3

**Published:** 2016-06-24

**Authors:** Galina N. Pochukalina, Nadya V. Ilicheva, Olga I. Podgornaya, Alexey P. Voronin

**Affiliations:** Institute of Cytology, Russian Academy of Sciences, St Petersburg, 194064 Russia; Saint Petersburg State University, St Petersburg, 199034 Russia; Far Eastern Federal University, Vladivostok, 690950 Russia

**Keywords:** Mouse oogenesis, Morphology, Karyosphere, Nucleolus-like body, Immunofluorescence

## Abstract

**Background:**

During the final stages of oocyte development, all chromosomes join in a limited nuclear volume for the final formation of a single complex chromatin structure – the karyosphere. In the majority of mammalian species, the chromosomes surround a round protein/fibrillar body known as the central body, or nucleolus-like body (NLB). Nothing seems to unite the inner portion of the karyosphere with the nucleolus except position at its remnants. Nevertheless, in this study we will use term NLB as the conventional one for karyosphere with the central body. At the morphological level, NLBs consist of tightly-packed fibres of 6–10 nm. The biochemical structure of this dense, compact NLB fibre centre remains uncertain.

**Results:**

The aim of this study was to determine which proteins represent the NLB components at final stages of karyosphere formation in mouse oogenesis. To determine this, three antibodies (ABs) have been examined against different actin epitopes. Examination of both ABs against the actin N-end provided similar results: spots inside the nucleus. Double staining with AB against SC35 and actin revealed the colocalization of these proteins in IGCs (interchromatin granule clusters/nuclear speckles/SC35 domains). In contrast, examination of polyclonal AB against peptide at the C-end reveals a different result: actin is localized exclusively in connection with the chromatin. Surprisingly, no forms of actin or topoisomerase II are present as components of the NLB. It was discovered that: (1) lamin B is an NLB component from the beginning of NLB formation, and a major portion of it resides in the NLB at the end of oocyte development; (2) lamin A undergoes rapid movement into the NLB, and a majority of it remains in the NLB; (3) the telomere-binding protein TRF2 resides in the IGCs/nuclear speckles until the end of oocyte development, when significant part of it transfers to the NLB.

**Conclusions:**

NLBs do not contain actin or topo II. Lamin B is involved from the beginning of NLB formation. Both Lamin A and TRF2 exhibit rapid movement to the NLB at the end of oogenesis. This dynamic distribution of proteins may reflect the NLB’s role in future chromatin organization post-fertilisation.

## Background

The mammalian oocyte nucleus or germinal vesicle (GV) exhibits a unique chromatin configuration that is subject to dynamic modifications during oogenesis. This process of epigenetic maturation is critical in conferring the female gamete with meiotic as well as developmental competence. In spite of its biological significance, little is known concerning the cellular and molecular mechanisms regulating large-scale chromatin structure in mammalian oocytes [1].

The epigenetic maturation morphologically appears to be the result of all chromosomes of the gametocyte joining in a limited nuclear volume with final formation of a single complex chromatin structure – the karyosphere. The karyosphere was named and first described by Blackman [2], who observed that the chromosomes in the spermatocytes of millipedes (Chilopoda) join to form a knot. The karyosphere is a form of chromosomal apparatus that sometimes exists for long periods of time within the oocytes of many animals, from hydra to higher vertebrates [3]. The term “karyosphere” has been suggested to designate the complex of a former nucleolus (also referred to as NLB), adjacent chromatin, and the adjacent nuclear bodies (including IGCs) in human GV oocytes [4]. However, although lampbrush chromosomes (which often precede karyosphere formation) have been discussed in numerous studies, karyosphere formation has received considerably less attention. The active state of the nucleus is succeeded by a decrease in the transcriptional activity of chromosomes and nucleoli, and the accumulation of chromosomes into a karyosphere. It is thought that karyosphere formation is the result of chromosomal inactivation in respect of RNA synthesis [5]. The morphological appearance of the karyosphere varies in the animal kingdom, though two main plans become evident: (1) karyosphere formation is paralleled by the appearance of newly-formed capsule-shaped structure around the chromosomes – karyosphere capsule (KC); (2) the chromosomes surround the round protein/fibrillar body – the central body [6] or nucleolus like body (NLB) [3]. It is generally assumed that the KC represents a specialized component of the oocyte nuclear matrix (NM) supporting the chromosomes of large GVs [5].

Sequential changes occurring in chromatin organization during folliculogenesis in mice has been described as the formation of a perinucleolar chromatin rim in the GV [7]. In the case of mouse oogenesis, other terms have been utilised to describe the changes. Chromatin in developing mouse oocytes is initially found decondensed, in a configuration termed the non-surrounded nucleolus (NSN). Subsequent growth leads to chromatin becoming progressively condensed, forming a heterochromatin rim in close apposition with the nucleolus remnants – thus acquiring a configuration termed surrounded nucleolus (SN) [3].

The oocytes’ transcriptional reduction is followed by the transformation of the nucleolar structure; the nucleolus gradually loses all of its classical components [8, 9], transforming into the structure known as a NLB. The NLB with chromatin surrounding the central body represents feature of many mammalians, including humans [3, 4, 8, 10]. Recently, several reviews of large-scale chromatin organization in mammalian oocytes have been published [11, 12].

Nothing seems to unite the inner portion of the karyosphere with the nucleolus except position at its remnants. NLB does not exhibit an argentophilic reaction [13]. The fibrillar component of the NLB central body consists of acid proteins. At the morphological level, NLB consists of tightly-packed fibres of 6-10 nm [3]. Some nucleolar proteins have been observed to move from the NLB into tiny granules at its periphery, and then in the nucleoplasm [3]. Few proteins, which are considered markers for the different nuclear compartments, have been found at the NLB periphery or in its vacuole: coilin, polymerase II, and splicing factors [3, 9]. Recently, the following nucleolar proteins have been discovered in the dense fibre centre of NLB, under special treatments: UBF, fibrillarin, NPM1, C23 [14, 15], NPM2 [16], and RPL26 [15]. This study investigates the presence of alternate protein components of the NLB.

The antibodies used were based on the assumption that the NLB could be a specialized component of the oocyte NM in the same manner as the karyosphere capsule. F-actin was revealed as a basic component of the KC in several insects [17–19] and also in frogs [20]. It has been shown that actin also has a crucial role in the maintenance of oocyte nuclear architectonics, and its depolymerization leads to a collapse of nuclear structures [21, 22].

One of the best-characterized extrachromasomal protein bodies, IGCs, often abut the mouse NLB [23]. IGCs are suggested as one of the most universal and evolutionarily-conserved nuclear domains [24, 25]. They primarily represent nuclear storage sites for pre-mRNA splicing factors [3, 26, 27], though extensive studies conducted during the past two decades have introduced the notion that IGC functions are broader than initially thought; these domains are involved in many other nuclear processes directly connected with gene expression and nuclear architecture [3, 25], and could participate in NLB formation.

Topoisomerase II (topo II) was one of the first NM proteins to be identified [28, 29]. Type II topoisomerases are archetypal nucleic acid remodelling enzymes that, using ATP as a cofactor, can varyingly add or remove DNA supercoils and either form or unlink DNA tangles [30]. Several studies on vertebrate systems indicate that this enzyme plays a role in the shaping of mitotic chromatin: topo II is the main factor in chromosome condensation, and represents a component of the chromosome core [31]. Topo II could be involved in the condensation process when chromatin is highly condensed around the NLB.

The nuclear lamina consists of lamins, which belong to type V intermediate filament proteins [32]. Lamins are categorized into two types, A and B, based on their biochemical and sequence characteristics. Small, yet significant fractions of both A- and B-type lamins are also present throughout the nuclear interior during interphase. Some of these internal lamins may be nascent proteins that were recently transported from the cytoplasm and in preparation for assembly into the lamina [33]. Several independent lines of experiments suggest that the A- and B-type lamins form separate filamentous networks in the lamina of somatic cells. High resolution confocal microscopy suggests that each type of lamin forms a distinct meshwork structure with a relatively small number of points of colocalization in somatic cells [34]. The same is true for oocytes. Based on electron microscopy, it is evident that overexpressed lamin A and lamin B2 form different types of filaments in separate intranuclear compartments of Xenopus oocyte nuclei. Lamin B2 formed irregular, wavy filaments associated with intranuclear membrane structures induced by the expression of lamin B2. In contrast, lamin A formed thick, multi-layered assemblies of filaments closely associated with the endogenous lamina formed by lamin B3 [35]. This was the reason for the utilisation of antibodies against both lamin A and lamin B.

The nuclear lamina and the internal nuclear matrix (NM) are two parts of the NM preparations [36]. The NM is a network dispersed throughout the nucleus, which is operationally defined as being resistant to high salt or detergents, i.e. insoluble [37]. Since it is associated with protein machinery for transcription, RNA splicing, and DNA replication, NM is believed to play a fundamental role in the organization of these processes [36, 38]. We will use term “nuclear matrix” (NM) in the current paper, as a NM preparation was initially used for mouse TRF2 isolation.

Telomeric binding factor 2 (TRF2) was discovered in the outer fraction of the NM, i.e. nuclear lamina [39], and was originally isolated from the nuclear envelope of frog oocytes [40]. The attachment of telomeres to the nuclear envelope in meiosis, and the resulting “bouquet” formation are thought to promote proper chromosome pairing via the concentration of chromosome attachment sites within a limited region of the nucleus [41]. This attachment undoubtedly occurs in germ cells during the central meiotic phases [42], though latter frog oocyte chromosomes are packed into a tight karyosphere with KC. Oocytes were collected at the stage when chromosomes are separated from the envelope, but it was assumed that the protein of interest remained associated with it. As a result, a membrane-associated telomere-binding protein (MTBP) was discovered. The protein exhibited binding specificity to telomeric DNA, and anti-MTBP antibodies (AB) were raised in guinea pig [40, 43, 44].

Two vertebrate proteins which bind to double-stranded telomeric DNA have been described: TRF1 [45, 46] and TRF2 [47, 48]. Six core proteins: TRF1, TRF2, TIN2, POT1, TPP1 and Rap1, form the telosome or shelterin complex, regulating telomere structure and function [49]. Both TRF1 and 2 contain a specific Myb-related protein motif - telobox peptide [47]. It is assumed that TRF2’s main role is to protect telomeres from DNA repair activities, which would prevent chromosomal aberrations involving chromosome ends such as end-to-end fusion [50, 51].

TRF2 is tightly bound to the nuclear membrane in frog oocyte nuclei [43], in the nuclear envelope and its remnants in mouse cells [39]. Lamin B was used as the protein marking the nuclear envelope remnant. Lamin B (a protein associated with the nuclear envelope remnants during mitosis) [52], and TRF2 colocalised as demonstrated by the double AB labelling. TRF2 antibodies were used to check protein position with respect to the NLB.

The aim of this study was to determine which proteins compose the NLB’s central body during the final stages of karyosphere formation in mouse oogenesis.

## Methods

### Oocytes

Female Balb/C mice were purchased from the Rappolovo Breeding Centre of the Russian Academy of Medical Sciences (Rapplolvo, Russia). Preovulatory oocytes from the antral follicles of sexually mature mice (one to two months of postnatal development) were used. The cumulus-enclosed oocytes were collected from ovaries by gentle puncturing of antral follicles with a needle in 4 % formaldehyde, freshly-prepared from paraformaldehyde in a phosphate-buffered saline (PBS) solution to prevent the resumption of meiosis. Oocytes were subsequently incubated in 0.1 % Triton X-100 in phosphate-buffered saline (PBS) for 10 min. Overall, approximately 300 oocytes were used in this study, and 10 oocytes were used for each treatment. All experimental treatments contained oocytes from three to five different mice. All experiments were repeated at least three times.

### Antibodies

The following primary antibodies (ABs) were utilized in this study: mouse monoclonal antibody (mAb) against non-snRNP splicing factor SC35 (Sigma; cat. no. S4045; dilution for immunofluorescence (IF) 1:50); mouse mAb against lamin A (Abcam; cat. no. ab8980; dilution for IF 1:100, for Western blot (WB) 1:500); rabbit polyclonal antibody (pAb) against TRF2 (Abcam; cat. no. ab 4182; dilution for IF 1:100, for WB 1:2000); rabbit pAb against topo II (Sigma; cat. no. AV04007; dilution for IF 1:200); rabbit pAb against a synthetic peptide conjugated to KLH derived from within residues 400 - 500 of Mouse lamin B1 (Abcam; cat. no. ab16048; dilution for IF 1:100, for WB 1:3000). ABs against different actin parts: mouse mAb against actin N-end (amino acid residues 50-70) (Millipore; cat. no. MAB1501R; dilution for IF 1:50); rabbit pAb against actin N-end (the first nine amino acid residues of the N-terminal region of actin) (Sigma; cat. no. A2103; dilution for IF 1:200); rabbit pAb against actin C-end (peptide of 11 amino acid residues AGPSIVHRKCF) (Sigma, cat. no. A2066, dilution for IF 1:200). It is known that AB MAB1501R reacts with all actin isoforms on immunoblot [53] and AB PAB A2066 was successfully used in immunoblot and immune-gold electron microscopy [21].

The antibody mixture was used for double staining of oocyte preparations [54]. Secondary antibodies were the Alexa-488 or Alexa-568 conjugated goat anti-mouse, goat anti-rabbit, rabbit anti-mouse immunoglobulins (IgGs) (Molecular probes; dilution 1:200). Secondary antibodies for Western blot were the anti-mouse IgG (whole molecule)-alkaline phosphatase antibody produced in goat (Sigma, cat. no. A3562) and the anti-rabbit IgG (whole molecule)-alkaline phosphatase antibody produced in goat (Sigma, cat. no. A3687; dilution 1:10000).

### Immunoblotting

The method for mouse liver nuclei isolation has already been described [55, 56]. Briefly, liver tissue was homogenised in ten volumes of pH 7.5 solution containing 0.32 M sucrose, 25 mM Tris-HCl, 5 mM MgCl_2_, and 1 mM PMSF. After centrifugation at 1000 rpm, raw nuclei were loaded onto a 2 M sucrose cushion and pelleted at 100,000 g (+4^0^ C) for 40 min. The preparations were checked for purity by phase-contrast microscopy, and the pure nuclei were used for Western blot to inspect the ABs. SDS-PAGE was conducted as described in a previous study [57]. The products were transferred to immobilon polyvinylidene fluoride transfer membrane (Millipore, Hertfordshire, UK) by Electroblot (BioRad Lab Ltd, Hertfordshire, UK) at 50 mA in an electrophoresis buffer containing 10 % ethanol. Blocking was conducted using 5 % skimmed milk for 1 hr in PBS (137 mM NaCl, 2.7 mM KCl, 10 mM Na_2_HPO_4_, 1.76 mM KH_2_PO_4_, pH 7.4) with 0.05 % Tween 20 (Sigma). This basic solution was used for all AB experiments. The first ABs, appropriately diluted in PBS-Tween buffer, were applied overnight at +4 °C. After washing with PBS-Tween, the blots were incubated with either anti-rabbit or anti-mouse alkaline phosphatase for 1 hr at room temperature, then stained with NBT-BCIP in a solution of 50 mM Tris-HCl (pH 9.5), 5 mM MgCl2, and 100 mM NaCl for around 30 min. Alkaline phosphatase added without first AB gave no staining.

### Immunofluorescence/confocal microscopy

Indirect immunofluorescent cytochemistry was conducted on total preparations of isolated oocytes using the method described in detail in previous papers [9, 58]. The incubation of the first antibody solution was conducted overnight in a moist chamber at 4 °C. After rinsing in PBS, the preparations were incubated with secondary antibodies for 1.5 h at room temperature. After rinsing in PBS, the preparations were additionally stained for 1 min with DAPI (Molecular probes; dilution 1:1000) to reveal DNA, and mounted in Vectashield medium (Vector Laboratories). Preparations were analysed in a Leica TCS SP5 confocal microscope equipped with argon (488 nm) and helium-neon (543 and 633 nm) lasers at 40x objective (NA 1.25). Merged images were obtained using ImageJ 1.37a software (National Institutes of Health).

### Immuno-gold electron microscopy

Oocyte fixation and embedding for electron microscopy were performed using a routine technique [4]. Oocytes were prefixed for 1.5 h in a solution containing 4 % formaldehyde (Ted Pella, Redding, Calif., USA) and 0.5 % glutaraldehyde in PBS, then fixed overnight in 2 % formaldehyde at 4 °C. After rinsing in PBS containing 0.05 M NH_4_Cl (Sigma) and subsequent dehydration in an ethanol series, oocytes were embedded in medium grade LR White resin (Polyscience, Warrington, Pa., USA). Ultrathin sections were incubated for 10 min in a blocking buffer containing 0.5 % fish gelatin (Sigma) and 0.02 % Tween-20 (Sigma) in PBS (pH 7.4). Sections were then incubated in the primary antibody solution overnight in a moist chamber at 4 °C. After rinsing in PBS containing 0.1 % fish gelatin and 0.05 % Tween-20, the sections were incubated with secondary goat anti-mouse and goat anti-rabbit IgGs conjugated with 10 nm gold particles (Electron Microscopy Sciences, USA). As a control, additional sections were incubated only in secondary antibodies. Ultrathin sections were contrasted with 1 % uranyl acetate-water solution and examined in a Carl Zeiss Libra 120 electron microscope operated at 80 kV. Magnification was inserted to the images automatically. The figures were prepared in Adobe Photoshop (Adobe Systems).

## Results

### Immunoblotting

**Fig. 1:**
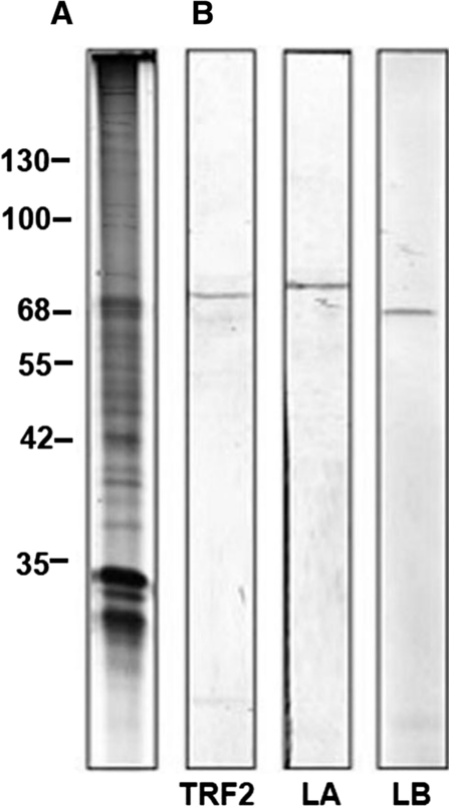
SDS-PAGE (**a**) and Western blot (**b**) of mouse liver cells nuclei. A - 8 % SDS-PAGE stained with Coomassie Brilliant Blue; 1 – molecular masses of the marker proteins are at the left in kDa. B – Western blot; the antibodies are indicated under each lane (TRF2, LA – Lamin A, LB – Lamin B). Working dilutions are given in Methods.

The main antibodies (ABs) used in this study were examined using Western blot (Fig. 1). ABs against TRF2 do not have cross-reactivity with TRF1. The band correspond to the apparent molecular mass (Mr) of 70 kDa while TRF1 Mr is 60 kDa [43, 59]. ABs against Lamin A and Lamin B also do not react with the opposite type. Therefore, these ABs could be used to trace their corresponding proteins. AB (mAb) against splicing factor SC35 was successfully utilized in previous studies to trace IGCs in somatic mouse cells [60]. All three ABs against actin stain one band of ~42 kDa on Western-blot, though their advantage is the possibility to recognize different actin epitopes, which is beyond Western blot’s resolution. Western blot could not distinguish which domain is stained on immunoblot. Therefore, reliance existed upon commercial descriptions or the existing literature published (see Material and Methods).

### Cumulus cells

**Fig. 2:**
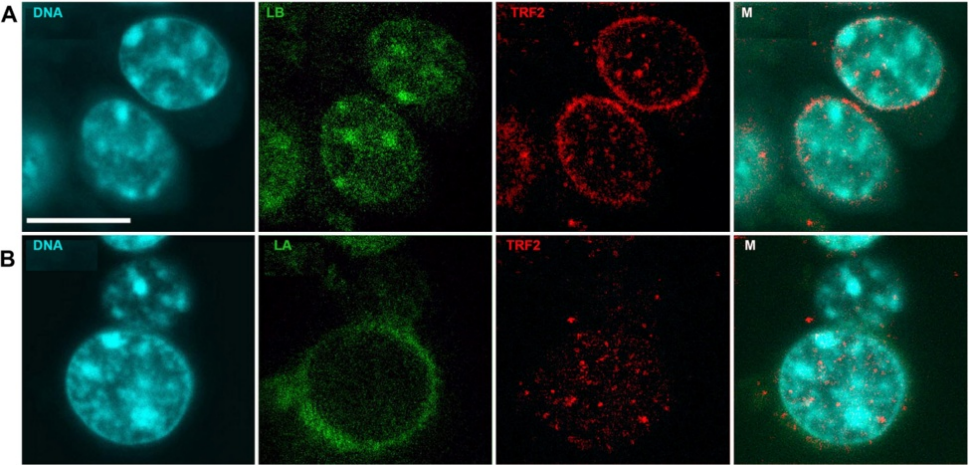
Cumulus cells stained with Lamin B (**a**) and Lamin A (**b**). The staining is indicated on each panel: DNA - DAPI; Lamin A (LA) and Lamin B (LB); TRF2 is red. Merged image is marked (M). Bar 10 μm for all images

Mouse oocytes grow in multilayer follicles composed of cumulus cells, which could always be found in preparations. Cumulus cells were used as an example in order to trace the typical staining of somatic cells. It is evident that cumulus cells’ nuclei possess prominent chromocentres, as is characteristic for somatic mouse cells [61]. DNA staining highlighted the alignment of chromocentres mainly at the border of the nuclei, and a number of them were located in the nuclei interior. Chromatin arranged into distinct radial zones which could be determined after DAPI staining. Ordered radial alignment of chromocentres has been observed for chickens [62], and similar arrangement could be traced on some of our images (Fig. 2, DNA).

Lamins A and B represent necessary components of the somatic cells’ nuclear envelope (NE), though staining with ABs is different. Lamin A mostly underlines the NE (Fig. 2, LA), while lamin B gravitates toward chromocentres (Fig. 2, LB). Both lamin types are known for their input in gene silencing [63], but lamin B1 is mainly immobile. This suggests that the internal B-type lamins are tightly associated with other relatively immobile structures such as heterochromatin [34].

TRF2 locations in somatic [44] and spermatogenic [64, 65] cells have been determined. It has been suggested that TRF2 possesses intimate connections with the nuclear envelope [66]. Mouse chromosomes are telocentric, with the centromere located adjacently to one of the two telomeres. The telomeric and centromeric regions of chromosomes form the heterochromatic material, and are therefore involved in chromocentre formation. The TRF2 location in cumulus cells corresponds to the one previously observed: a portion of the TRF2 label is at the NE and another portion is at the rim of the inner chromocentres (Fig. 2, TRF2).

As a result, it is evident that ABs work at immunoblot and exhibit the expected positions in somatic cells.

### Oocytes’ stages classification

**Fig. 3:**
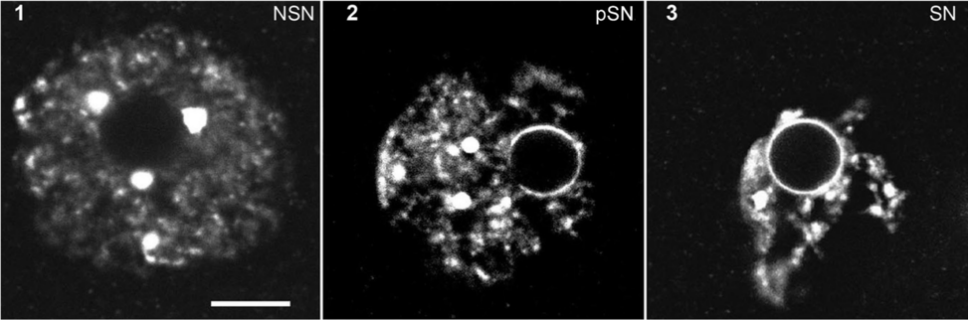
Three types of chromatin configurations in mouse oocytes’ nuclei. The confocal single z-sections of DNA staining with DAPI are shown in greyscale. Stage 1 - the NSN-type oocytes (Non Surrounded Nucleolus) show a decondensed chromatin configuration; Stage 2 - the Intermediate (Int) oocytes show a less condensed chromatin and a partial ring of chromatin around the NLB; stage 3 - the SN-type oocytes (Surrounded Nucleolus) show a highly condensed chromatin with a ring around the NLB central body. Bar 10 μm

The oocyte nucleus is subjected to important and relatively rapid nuclear architecture modifications during late growth stages. Chromatin is visible on the DAPI stained images, and stages of oocyte development could be determined from the NLB position – being central at the stage 1 (Fig. 3, 1). The characteristic feature of oocyte development, the karyosphere traced by the NLB moves toward the NE at stage 3 from its central position at stage 1 (Fig. 3). The final steps of the karyosphere formation are divided into 3 stages for descriptive purposes, and follow the classification published [67, 68]. In 1^st^ stage oocytes, euchromatin exhibits a decondensed configuration, with heterochromatin aggregates distinguished as chromocentres (Fig. 3, 1). In intermediate stage 2 (Fig. 3, 2) the karyosphere displays a mixed configuration in between the 1^st^ and 3^rd^ stages, with decondensed chromatin in the nucleoplasm and only a partial ring of chromatin around the NLB. At stage 3 (Fig. 3, 3), chromatin is highly-condensed and forms a ring around the NLB.

### Nuclear actin

In somatic cells, actin filaments form part of a large, viscoelastic structure in the nucleoplasm, and may act as scaffolds which help to organize nuclear contents [69]. It is reasonable to assume that structural proteins could be NLB components. Therefore, three types of ABs against actins’ different domains were used to trace the different forms of actin which can exist within the oocyte nucleus.

**Fig. 4:**
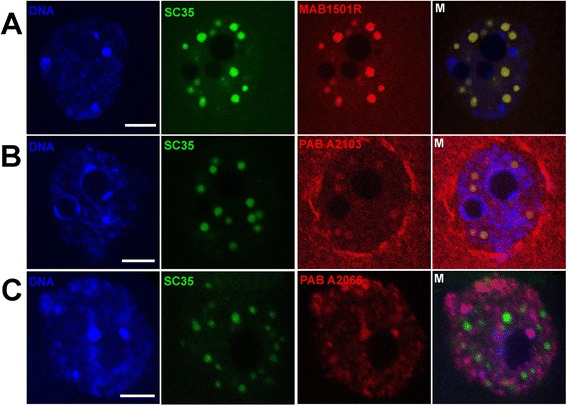
Double immunofluorescence of oocytes’ nuclei with SC35 AB (green) and three ABs against actin (red). **a** – 1^st^ stage oocyte stained with MAB1501R (amino acids residues 50-70 at N-end); (**b)** – 2^nd^ stage oocyte stained with PAB A2103 (the first 9 amino acids residues at N-end); (**c**) - 1^st^ stage oocyte stained with PAB A2066 (11 amino acids residues at C-end). M – merged images. DAPI staining is in blue (DNA). Bar 10 μm for all images

During all stages of oocyte development, both ABs against actin N-end exhibited similar results: spots inside nucleus (GV). Double staining with AB against SC35 and actin revealed the colocalization of these proteins in IGCs (Fig. 4a, b). This perfect correspondence is visible in the case of AB MAB1501R against 20 amino acid residues at actin N end (Fig. 4, a). Polyclonal AB against the first 9 amino acid residues at the actin N-end also reveals actin in IGCs, though nuclear envelope is also underlined (Fig. 4, b). Some diffuse staining around the nucleus in the cytoplasm was also observed. In contrast, polyclonal AB against peptide at the C-end reveals a different result: actin exclusively followed chromatin detected by DAPI staining. IGCs contain only the marker protein SC35. AB A2066 reveals only the actin co-localized with the chromatin. Both types of the chromatin are stained: the diffuse part and the one parked in chromocentres. This is clearly visible in the merged images (Fig. 4, c, M). These ABs recognize nuclear actin exclusively, without any staining in the cytoplasm or in the space surrounding nuclei (Fig. 4, C vs. B).

The difference in actin AB staining suggests that different forms of monomeric (oligomeric) actin exist in oocyte nuclei [70], but not a single form of actin is involved in NLB formation.

### Topo II

Topo II was identified within the chromosome scaffold fraction. Immunostaining of unextracted chromosomes and in-vivo observations have confirmed the existence of an axial core distribution in native metaphase chromosomes for topo II [71].

**Fig. 5:**
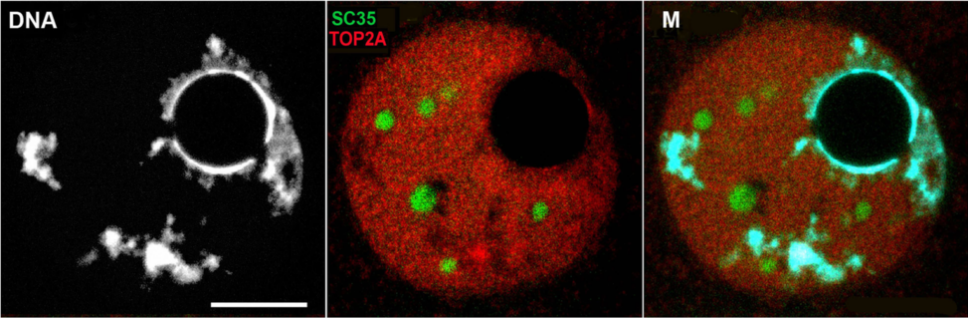
Stage 3 oocyte nucleus double stained with AB against SC35 (green) and Topo II (red). DAPI staining is in grayscale (DNA); M – merged image. Bar 10 μm

AB against topo II (Topo2A) were used to check its position in the oocyte GV. In spite of chromatin being highly condensed in the late oocytes’ nuclei, Topo II distribution differs from mitotic chromosomes in that no recruitment of Topo II to chromatin exists at the rim of NLBs (Fig. 5, DNA). Topo II displays a dotted pattern throughout the nucleoplasm with unlabelled IGCs. Additionally, NLB does not contain topo II at all stages; Fig. 5 represents the 3^rd^ stage oocyte, and staining of 1^st^ and 2^nd^ stage oocytes appears similar. The enrichment of the label rather follows the condensed chromatin, yet does not coincide with it (Fig. 5).

### Lamins

**Fig. 6:**
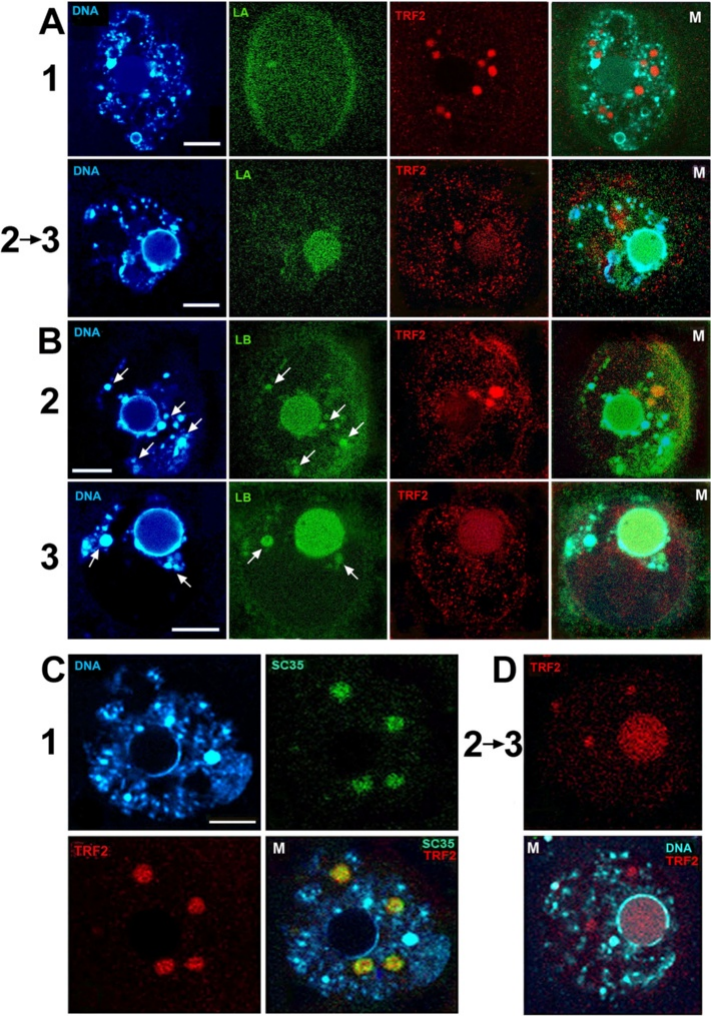
Double immunofluorescence of oocytes nuclei with TRF2 AB (red) and other ABs (green). Oocyte stages are indicated at the left for each panel (**a**, **b**, **c**, **d**). A – AB against Lamin A; B - AB against Lamin B. 1^st^ image in each row – DAPI (DNA), M – merged images. C – staining is indicated on each image; D – TRF2 and DAPI staining of the nucleus progressing from stage 2 to stage 3. Bar 10 μm for all images

**Fig. 7:**
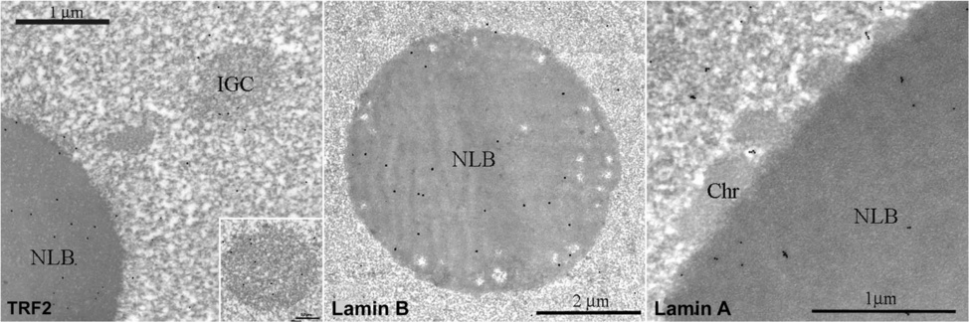
Immunogold labeling of TRF2, Lamin B and Lamin A in mouse oocytes. TRF2 - TRF2 is component of NLB at stage 3, IGCs contain few grains of label, while at the previous stages IGCs are heavily stained (insert); Lamin B - Oocyte NLB of 1^st^ stage is enriched with Lamin B. Note loose NLB structure and absence of condensed chromatin blocks on his NLB periphery, which is typical for 1^st^ stage NLB; Lamin A - Oocyte NLB of 3^d^ stage is enriched with Lamin A. Note blocks of condensed chromatin (Chr) at the NLB surface. Bars are indicated for each image

Lamins were the subsequent proteins to be traced. Lamins represent the main proteinaceous components of the nuclear envelope. Lamin B is present in the NLB from the 1^st^ stage though it always marks the chromocentres remnants (pointed by arrows, Fig. 6, b; Fig. 7, Lamin B). Lamin A also comes to the NLB from 1^st^ to 3^rd^ stage (Fig. 6, a). It appears as though a higher level of lamin B than lamin A remains at the NE. The NE should retain its integrity, and lamin B better suits this purpose. There are cells without lamin A, but no cells without lamin B type. T-cells and B-cells express only B-type lamins; undifferentiated human and mouse embryonic stem (ES) cells lack lamins A/C, but express lamins B1 and B2 [72]. B-type lamin is expressed throughout embryogenesis, whereas lamin A and lamin C are not expressed until the tissue differentiation stage of development [33]. It is visible that some of lamin A is stored in the NLB to be used, likely in early embryogenesis (Fig. 6, a).

Chromocentres with low transcriptional activity are visible by intensive DAPI staining (Fig. 2, DNA). Namely, the chromocentres are stained with lamin B AB in cumulus cells (Fig. 2, a). The same tendency is visible in oocytes: lamin B gravitates toward chromocentres, while lamin A never marks them (Fig. 6, a, b). Together, these images suggest a difference in lamin type distribution, and the involvement of lamin B in chromocentres formation. Both lamin types are involved in NLB formation (Fig. 6, a, b; Fig. 7). Lamin A exhibits rapid migration from the NE to the NLB between the 1^st^ and 3^rd^ stages of oocyte development.

### TRF2

TRF2 is not connected with telomeres during all 3 final stages of oogenesis. It is established that most heterochromatic regions with telomeres and centromeres move to the rim of the karyosphere and NLB at stage 3, and coincide with chromocentres at previous stages [68]. TRF2 was not observed to coincide with chromocentres (Fig. 6). This result is unsurprising, as TRF2 is known to be separated from telomeres, and attached to the nuclear envelope in frog oocytes. In the diplotene stage, TRF1 remains connected with telomeres in chromatin, whereas TRF2/MTBP does not [43]. Its membrane location has been established by biochemical and morphological observations in mouse cells as well [44, 64]. In mouse oocytes, TRF2 at stages 1 and 2 resides in SC35 domains (Fig. 6, c, d) and moves rapidly to the NLB at stage 3 (Fig. 6).

It was discovered that TRF2 is an IGCs resident at certain stages of oocyte maturation. The correspondence of TRF2 and SC35 labels inside IGCs is not perfect (Fig. 6, c, d); both present in a dotted pattern, though dots abut to each other rather than overlay. SC35 domains are the last TRF2-containing compartment prior to its relocation to the protein central body (Fig. 6, d). Thus, the behaviour of some proteins remains highly dynamic even during last stages of oocyte maturation. Y14, the core protein of the exon junction complex, was the first to have its transient position in IGCs confirmed by the gene engineering approach [73]. Now, TRF2 exhibits the same tendency: it moves quickly from IGCs to NLBs.

Immuno-gold electron microscopy (EM) has been conducted to confirm proteins’ presence in NLB (Fig. 7). Lamin A is included in the NLB at stage 3 (Fig. 7, Lamin A), and lamin B is the NLB component from the beginning of its formation (Fig. 7, Lamin B). The TRF2 AB also exhibits heavy NLB labelling, and few grains are present in IGCs – though IGCs did contain TRF2 at previous stages (Fig. 7, TRF2, insert). Mouse oocyte IGCs have been described at the EM level [23]. Large mouse preovulatory oocytes contain 10–20 roundish bodies, which are clearly defined morphologically (1–4 μm in diameter), scattered throughout the nucleoplasm. Mouse oocyte IGCs display several ultrastructural peculiarities. Their granules are 10–15 nm, which is slightly smaller than the typical 20-25-nm interchromatin granules of somatic cells [3]. It is likely that the increase in size and acquisition of a IGC’s roundish form in karyosphere and NLB-containing oocyte nuclei is the consequence of oocyte transcription inactivation. Similar morphological changes occur in somatic cells treated with transcription inhibitors [3]. TRF2 belongs to the fibrillar IGCs’ component when included in these structures. As a result, immune-gold labelling confirms TRF2 movement from IGCs to the NLB, and the involvement of lamin A and lamin B in NLB formation.

## Discussion

### Proteins absent in the NLB

#### Actin

The function and form (monomer, filament, or noncanonical oligomer) of nuclear actin is highly-contested, and its localization and dynamics are largely unknown. However, a break-through occurred using the gene engineering approach [69]. A set of fluorescent nuclear actin probes have been designed, constructed, and validated to visualize nuclear actin monomers and filaments in live somatic cells (human U2OS osteosarcoma). The probes’ construction allows the distinguishing of monomeric from polymeric actin. It is shown that probes which bind to monomeric actin are concentrated in IGCs. Filamentous actin forms a set of punctate structures of uniform size. These structures are scattered throughout the interchromatin space, and are excluded from chromatin-rich regions. These observations argue against direct participation of the majority of canonical actin filaments in gene regulation or chromatin remodelling [69].

Filamentous actin exists in oocyte nuclei [21, 22], though it is hardly the only form of actin present. With the set of ABs used, the position of actins’ various forms were traced, and an attempt was made to determine the mechanism by which nuclear monomeric actin is contained in a unique microenvironment to regulate chromatin interaction – instead of supporting actin polymerization.

Actin consists of four subdomains labelled S1–S4. Subdomains S2 and S4 make up the pointed (-) end of actin, and subdomains S1 and S3 represent the barbed (+) end [74]. In classical actin polymerization, filament grows from its barbed end to where the pointed end of the next monomer attached. Both N-end and C-ends included in the S1 subdomain and epitope C4 (MAB1501R) are embedded in S1. S2 subdomain is involved in interactions with DNA-binding proteins [74].

Spontaneous actin polymerisation is inhibited by proteins that sequester actin monomers, such as profilin and thymosin b4 [70, 75, 76]. Profilin is the SC35 domain component in the mouse oocyte [77]. C-end is directly involved in profilin binding; therefore it could be expected that only the N-end is available for the interaction, and consequently, only ABs against N-end recognize the actin-profilin complex (Fig. 4, a, b). AB against the first nine amino acid residues of the N-terminal region of actin (PAB A2103) also recognizes short actin oligomers surrounding the nuclear envelope (Fig. 4, b). The first amino acids of actin N-end are not involved in interactions with other proteins, and could be free in oligomerization. In somatic cells, nuclear actin filaments form short scaffolds that interact with a viscoelastic structure abutting the nuclei membrane [69]. It is also possible that the same type of structure is recognized in the oocyte by A2103 AB (Fig. 4, b).

The opposite end of the actin monomer, S2 subdomain, is involved in interaction with DNAse and components of the chromatin remodelling complex. Actin alone was unable to bind DNA, while the INO80 chromatin remodelling complex with its actin–Arp module can bind DNA [70, 77]. In the context of the chromatin remodelling complex, nuclear actin has gained the ability to either interact directly with chromatin or regulate chromatin binding indirectly through conformational changes [78]. Antibody against the C-end (A2066) reveals that actin is in tight association with the oocytes’ chromatin. Staining does not depend on the chromatin state - whether it packed in chromocentres or relatively dispersed (Fig. 4, c). Similar results have been obtained on NPB (nucleolus precursor bodies) of early mouse embryos. Actin is not a component of NPB in either male or female pronucleus [79]. AB against actin C-end (A 2066, Sigma) reveals one of the actin forms in association with the rim of NPB while AB against actin N-end (A2103, Sigma) stain actin outside the nucleus [80]. The NPB and NLB are considered to be related; the content of these structures could be dynamic, especially after fertilisation. Nevertheless, actin is not found within these bodies.

#### TopoII

Topo II and SMC2, components of the condensing complex, were the first proteins identified in the mitotic chromosome scaffold. The main representatives within the scaffold fraction are Topo II [28, 31], condensins [31], and KIF4A [81, 82].

Previous studies have analysed the dynamic behaviour of major scaffold proteins in somatic cells, suggesting that several scaffold proteins are in fact very dynamic in relation to their presence on chromosomal structure. The most dynamic scaffold component is KIF4A (t. 2.5 s) [82], followed by topo II (t. 15 s) [83]. The expression of topo II-isoform increases during the late S phase, decreases at the end of the M phase, and is dramatically reduced in the G1/G0 phase of the cell cycle [84]. Then, an anti-Topo II-α antibody labels cells in the S, G2, and M phases of the cell cycle [71]. An oocyte, with its long diplotena, is at meiosis 1^st^ prophase when karyosphere formation occurs. Therefore, relatively prominent Topo2A staining can be observed. However, it is evident that topo II is not involved in chromatin condensation in this case (Fig. 5). No dramatic changes were observed in topo II location during oocyte maturation from the 1^st^ to 3^rd^ stages.

As a result, actin and Topo II do not represent protein components of the NLB.

### NLB proteins are dynamic at last stages of oocyte maturation: IGC versus NLB

The very term nucleolus like body (NLB) reflects its’ relation to nucleolus, so the ribosomal proteins were the first to check. While no nucleolar protein has been detected within the NLB mass by conventional immunocytochemistry, a protease digestion assay was applied to find putative presence of the nucleolar proteins in the NLB interior. The dynamic distribution of some of the proteins have been noticed. The ribosomal RPL26 protein was detected within the NLBs of NSN-type oocytes (1st stage) but is virtually absent from NLBs of SN-type oocytes (3d stage). Same is true for the ribosomal RNA (rRNA). Fluorescence in situ hybridization with oligonucleotide probes targeting 18S and 28S rRNAs shows that, in contrast to active nucleoli, NLBs of fully-grown oocytes are impoverished for the rRNAs, which is consistent with the absence of transcribed ribosomal genes in the NLB interior. Authors conclude that NLBs of fully-grown mammalian oocytes serve for storing major nucleolar proteins but not rRNA [15]. Even major nucleolar proteins such as UBF, fibrillarin, NPM1/nucleophosmin/B23, nucleolin revealed after proteinase K treatment show clear redistribution inside NLB between NSN-type (1st stage) and SN-type (3d stage) oocytes [15]. In the current study we discovered that lamin A, lamin B and TRF2 also exhibit dynamic distribution during last stages of oocyte development.

#### Lamins

In recent years, evidence has begun to accumulate regarding the association of A- and B-type lamins with different types of chromatin. It was assumed that A-type lamins are preferentially associated with gene-rich regions of active chromatin [34]. In somatic cells, lamin-A was discovered proximal to the nuclear membrane (Fig. 2, b). No active chromatin was present in the oocyte nucleus, and the staining with AB against lamin A belongs mainly to the nuclear envelope at stage 1 (Fig. 6, a). Lamin B1 appears to be primarily associated with the borders of regions with low gene density and low transcriptional activity. Genomic domains of 50 kb to 10 Mb in size are bounded by regions that are enriched in interactions with lamin B1 [85]. These domains, called lamin-associated domains (LADs), may represent the heterochromatin frequently observed as being closely opposed to the inner NE in many somatic cells. The morphological approach does not allow for the distinguishing of LADs, though chromocentres with low transcriptional activity are visible by intensive DAPI staining (Fig. 2, DNA). Namely, the chromocentres are stained with lamin B AB in cumulus cells (Fig. 2, 2) as well as in oocytes: lamin B gravitates toward chromocentres, while lamin A never marks them (Fig. 6, a, b). So, lamin B is involved in chromocentres’ formation in both cell types. Both lamin types are involved in NLB formation (Fig. 6, a, b; Fig. 7). The rapid migration of lamin A from the NE to the NLB between the 1^st^ and 3^rd^ stages of oocyte development coincides with the TRF2 movement.

#### TRF2

Telomere-membrane associations are often observed in morphological studies [86], and TRF2 is the good candidate for the responsibility of attaching the telomere-protein complex to the nuclear envelope, though its relationship to the lamins remains to be elucidated in detail. A-type lamins certainly affect both nuclear membrane and telomere dynamics [87].

In mice pachytene spermatocytes, membrane structures which abut the synaptonemal complex attachment sites contain TRF2. During spermiogenesis and in fully formed spermatozoa, TRF2 unexpectedly localized at the acrosomal membrane that is adjacent to the nucleus – apart from the expected TRF2 position at the nuclear periphery. Telomere distribution is not static in cultured cells throughout the cell cycle or during spermatogenesis. When telomeres are attached to the nuclear envelope, such as during synaptonemal complex formation, TRF2 is the member of the protein complex which appears to be responsible for telomere attachment [64].

The direct biochemical interaction between lamin A and TRF2 has been established [88]. A-type lamins have been widely discussed since the discovery that LMNA mutations or defective posttranslational processing of pre–lamin A causes the majority of human diseases (termed laminopathies) that are accompanied with shortened telomere lengths [89, 90]. A shift in telomere localization was observed in the absence of A-type lamins, suggesting an active role of A-type lamins in the positioning of telomeres. A-type lamins play a role in the maintenance of telomeres, though the molecular mechanisms remain unknown [91]. The findings of the current study contribute to the existing evidence of connection between lamin A and telomeres.

TRF2’s position in the oocyte NLB leads to the suggestion that TRF2 location could reflect preparation for fertilisation events. After fertilisation, the chromatin-surrounded NLB proceeds into second prophase mitosis, and should be assembled quickly in chromosomes. Chromosomes are not distinguished in the chromatin rim surrounding the NLB (Fig. 3; [68]). Likely, the NLB is a storage place for the proteins involved in chromatin orientation. NLB dissolved in 2^nd^ mitotic division helps to arrange the ring of chromosomes. The NLB’s important post-fertilisation role in future chromatin organization has been recently confirmed [14].

#### NLB structural role

Protein composition of the karyosphere central body, often referred to as NLB, especially of its’ dense fibrillar component, was obscure for a long time. Some nucleolar components were found in vesicles at the NLB periphery [9] or revealed after special treatments [14, 15]. The central body is formed at the former nucleolus place and some of its components can be captured, but they cannot be the major components. Nucleolar activity is absolutely suppressed in the NLB and de novo nucleolus activity is established while zygote NPB discarded [92]. Karyosphere central body is rather essential for proper chromosomes organization before fertilization. Lamins are one of the main components organizing high-order chromatin structures and were first to be found in the NLB, which seems to be consistent with the central body structural role. Fast move of TRF2 to the central body was unexpected; however, recently discovered direct interaction between TRF2 and lamin A explains the mechanism. TRF2 as the structural protein responsible for telomere-membrane attachment fits into the picture. Identification of lamins as one of the main fibrillar NLB components supports the idea that the NLB central body organizes chromatin before fertilization.

## Conclusions

It was discovered that: (1) Lamin B is a component of the NLB from the very beginning of its formation and major portion of it collected in NLB at the end of oocyte development; (2) lamin A undergoes rapid movement into the NLB, where the majority of it remains; (3) TRF2 resides in the IGCs up until the final stages of oocyte development, when a significant portion of it relocates to the NLB. Surprisingly, no forms of actin or topo II represented components of the NLB. Other proteins critical to chromatin organization are expected in NLB; this study reports the first findings of those present.

## Abbreviations

AB, antibody; BCIP, 5-bromo-4-chloro-3’indolylphosphate; DAPI, 4,6-diamidino-2-phenylindole; EM, electron microscopy; ES, embryonic stem cells; GV, germinal vesicle; IF, immunofluorescence; IGCs, interchromatin granule clusters; IgGs, immunoglobulins; KC, karyosphere capsule; LADs, lamin-associated domains; mAb, monoclonal antibody; Mr, molecular mass; MTBP, membrane-associated telomere-binding protein; NBT, nitro blue tetrazolium; NE, nuclear envelope; NLB, nucleolus-like body; NM, nuclear matrix; NPB, nucleolus precursor bodies; NSN, non-surrounded nucleolus; pAb, polyclonal antibody; PBS, phosphate-buffered saline; SN, surrounded nucleolus; TRF2, telomeric binding factor 2; WB, Western blot
